# Performance and Damage Study of Composite Rotor Blades under Impact

**DOI:** 10.3390/polym16050623

**Published:** 2024-02-25

**Authors:** Guorui Yu, Xiaobin Li, Wenjun Huang

**Affiliations:** AVIC China Helicopter Design and Research Institute, Jingdezhen 333001, China; yugr@avic.com (G.Y.); lxbin1990@163.com (X.L.)

**Keywords:** composite blade, failure criteria, finite element analysis, ballistic experiment

## Abstract

A military helicopter is easily attacked by bullets in a battlefield environment. The composite blade is the main lifting surface and control surface of the helicopter. Its ballistic performance directly determines the vulnerability and survivability of the helicopter in the battlefield environment. To study the ballistic performance of the composite helicopter blade, the damage characteristics of the impacted composite rotor blade are obtained by experiments. A numerical simulation model is established by applying Abaqus software to predict the blade ballistic damage. The three-dimensional progressive damage failure model is used to analyze the ballistic damage under the experimental conditions. The effectiveness and accuracy of the numerical simulation model are verified through a comparison with the experimental results. The ballistic damage of composite blades under three experimental conditions was investigated. The results show that the ballistic damage type of composite blade mainly includes delamination, fiber breakage, and foam collapse. The damage to the composite material at the position of bullet incidence is mainly local shear fracture, while the damage to the composite material at the exit position is mainly fiber tensile fracture. The ballistic damage size of the composite blade is closely related to the ballistic position, incident angle, and structure characteristics along the ballistic path. The larger the incident angle, the smaller the ballistic damage size of the blade. The greater the structural stiffness of the structure near the exit, the greater the damage size of the exit. The numerical simulation model presented in this paper can provide a reference for research on the ballistic performance of composite helicopter blades.

## 1. Introduction

Military helicopters are faced with different threats of ground weapons and air weapons in the battlefield environment. To avoid destructive destruction after the ballistic attack and improve the survivability of the aircraft, the military helicopter needs to have certain anti-ballistic capabilities. As an important part of a helicopter, the rotor blade is the main lifting surface and control surface of the helicopter, so the anti-ballistic capability of the blade should be paid more attention to.

Fiber-reinforced composite materials show excellent mechanical properties and better design ability compared with traditional metal. Composite rotor blades are widely used in modern helicopter design. Fiber breakage, delamination, and foam collapse of the composite rotor blades usually emerge under ballistic impact. And the bearing capacity of the composite material will be reduced, which poses a threat to helicopter safety. Therefore, the damage to the composite rotor blade under high-speed bullet impact will directly affect the helicopter. The composite rotor blades of common military helicopters, such as Black Hawk, V22, Apache, Comanche, NH90, and CH47, can resist one bullet of 12.7 mm or 23 mm bullets without affecting the safety of returning to the aircraft base [1,2,3,4,5,6,7].

Edward T. Keast [8] investigated the improvement method for the damage tolerance of the composite rotor blade. A variety of helicopter profile structures were designed, and the residual stiffness and performance of the blade after impact were analyzed. The damage and failure mechanisms of the impacted composite blade are studied by J. Aubry and P. Navarro [9]. A steel ball was used as an alternative to the bullet. Normal and oblique impacts were considered. Zach [10] established a macro-mechanical model of composite rotor blades to simulate bullet damage. Normal and lateral bullet damage were simulated. However, only unidirectional fiber composite was considered; the woven fiber composite and foam were neglected.

The composite rotor blade is essentially similar to a composite sandwich panel. In recent years, the high-speed impact characteristics of sandwich panels have been extensively studied. Four failure modes of composite foam sandwich structure, including delamination and buckling of composite panels, matrix cracking and fiber breaking, Degumming of panel/core interface, and Collapse of foam cores, mainly occur under impact load [11]. Wang H [12] proposed that the flexible matrix composites always had higher perforation resistance but larger deformation than the rigid matrix counterparts. Yashiro [13] and Bernard [14] studied the influence of layer sequence and angle on impact damage, and concluded that the delamination damage to the panels first occurs between layers with different mat angles. The size of the panels tends to increase gradually from the impacted side to the interface. Both the delamination damage between layers and the damage between fibers and substrates were related to the direction of the material. Lang [15] concluded that the interface adhesive layer of the panel/core was a weak link of the sandwich structure, which was easy to fail under impact load. This reduced the overall strength and stiffness of the sandwich structure. The effect of sandwich density changes on the high-speed impact properties of composite foam sandwich structures was investigated by Nasirzadeh [16]. The impact resistance of sandwich panels did not increase with increasing foam density. Mehran [17] reached a similar conclusion. In their study of high-speed impact energy absorption of polyurethane foam sandwich panels, it was found that the high-strength and high-modulus panels were the main energy-absorbing components, and increasing the density of foam sandwich did not significantly improve the energy-absorbing performance of the whole structure. Acanfora [18] proposed that the polypropylene honeycomb core and CFRP composite external skins sandwich configurations are characterized by better overall crashworthiness performances. Albayrak [19] showed that using rubber intermediate, the energy absorption ability of curved sandwich composite can be increase by a significant improvement.

The high-speed impact characteristics of curved composite sandwich panels were studied by Usta [20]. It was concluded that the curvature of the panels changed the energy absorption rates of sandwich panels, and the doubly curved sandwich panels had increased high-velocity impact resistance. Alonse [21,22,23] further studied the ballistic characteristics of composite foam sandwich panels. A continuum damage model was proposed. The energy absorption mechanism and the influence of different composite laminates on ballistic limits were analyzed. The damaged area of the front face skin was smaller than the damage produced in the rear face skin, and bending effects were notable in the latter, considering the largest fraction of the energy was absorbed by the out-of-plane.

Although the current research has been done to experiment with the ballistic performance of composite blades, only their anti-ballistic indicators have been verified, and no research has been conducted on the ballistic damage mechanism of composite blades. Comprehensive numerical research on the performance of the composite rotor blade has yet to be conducted. The high-speed impact analysis of the composite sandwich panels can reveal a certain mechanism of the impact resistance of the sandwich panels. However, it cannot reflect the effect of the impact position and impact angle of the bullet while investigating the full-size composite blade with a curved surface shape and stiffening ribs.

In order to study the ballistic performance of a full-scale composite blade, a type of composite rotor blade configuration with strong impact resistance is designed. The performance of composite blades under different ballistic impact conditions is studied by ballistic experiments. Based on this, a numerical simulation model considering the three-dimensional progressive damage failure model of the composite blade airfoil section is established. The simulation analysis of the damage of the composite blade under the condition of high-speed impact is carried out. The simulation results are compared with the experimental results to verify the accuracy of the presented numerical simulation model. And through the above analysis model, the damage mechanism of the composite rotor blade under bullet impact was studied. At the same time, the effects of ballistic position, incident angle, and ballistic path on ballistic damage were studied. The research results of this paper can provide a theoretical basis and reference for the anti-ballistic design and ballistic performance analysis of composite blades.

## 2. Specimen and Experiments

### 2.1. Design and Manufacture of the Experimental Specimen

**Figure 1:**
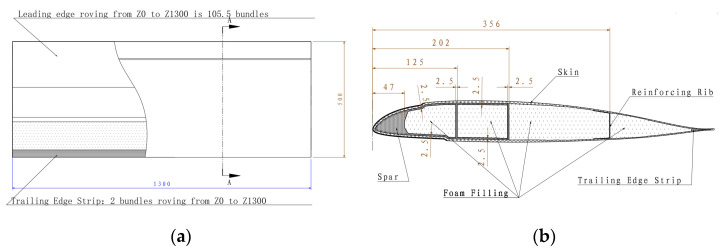
Composite blade airfoil section experiment specimen. (**a**) Schematic diagram of top view (unit: mm). (**b**) Schematic diagram of cross-section (unit: mm).

**Figure 2:**
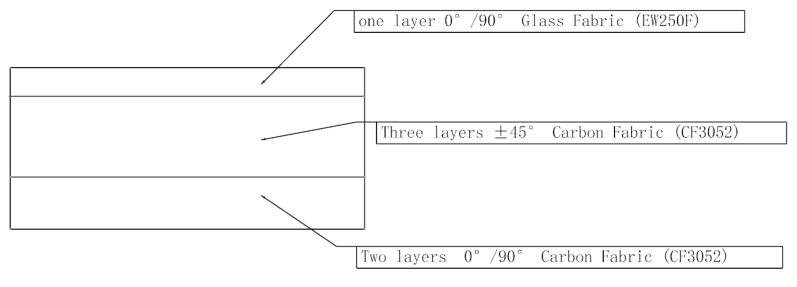
Schematic diagram of the skin layer.

The size and cross-section of the composite rotor blade airfoil section are shown in Figure 1. The spanwise length of the experiment specimen is 1300 mm. The chordwise length is 500 mm. The cross-section of the experiment specimen is a multi-closed cavity structure, mainly consisting of skin, spar, reinforcing rib, and foam filling. The skin and spar are the main load-bearing structures, which bear the centrifugal force and bending moment. The skin is mainly composed of carbon fiber fabric CF3502/epoxy and glass fiber fabric EW250F/epoxy (Beijing Institute of Aerial Materials, Beijing, China). The layer configuration and material properties of the skin are provided in Figure 2 and Table 1, respectively. The spar and the trailing edge strip consist of S_4_C10-800 glass fiber/epoxy prepreg tape (Beijing Institute of Aerial Materials, Beijing, China). Two closed box cavities in the D shape of the spar at the leading edge are formed. The material properties of S_4_C10-800 are shown in Table 1. The reinforcing rib consists of one layer of CF3502 and one layer of EW250F, which are laid in the rear edge of the mid-foam filling in a U shape. The foam filling selects polymethacrylimide (PMI) foam.

### 2.2. Experimental Process and Equipment

**Figure 3:**
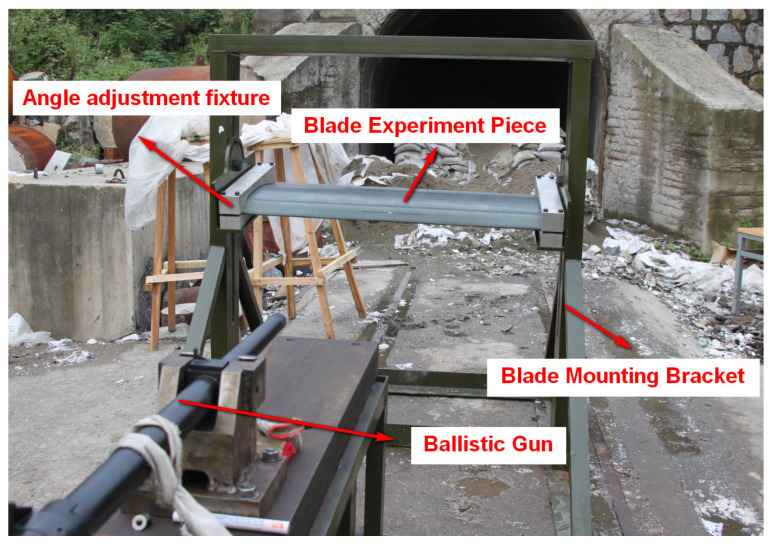
Test scheme and fixture.

**Figure 4:**
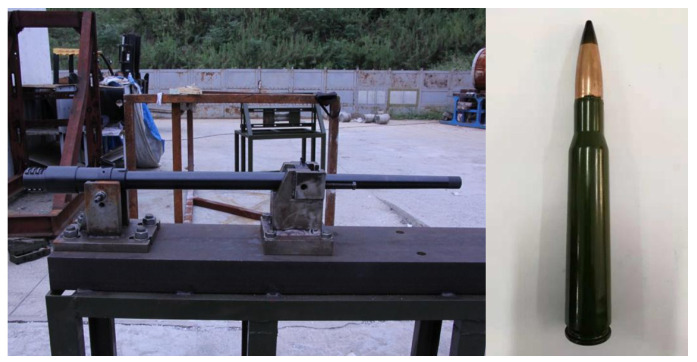
Ballistic gun and bullet.

As shown in Figure 3, the blade airfoil specimen is fixed on a steel structure mounting bracket arranged on the ground. Two angle adjustment fixtures are arranged on the fingernails, which are used for adjusting and fixing the blade airfoil specimen. Both fixtures help to change the ballistic impact angle relative to the blade airfoil specimen. As shown in Figure 4, the bullet used in the experiment is a 12.7 mm API. The 12.7 mm linear bore ballistic gun is applied to fire the bullet. A pair of light-screen targets are arranged on the ballistic line in order to measure the velocity of the bullet during the experiment process.

**Figure 5:**
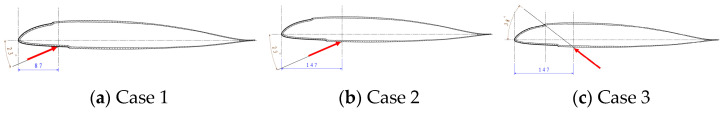
Cases of the projectile experiment (The red arrow represents the direction of the impact) (unit: mm).

In order to verify the ballistic performance of the composite blade airfoil specimen, three experimental cases were developed. The specific experiment conditions are shown in Figure 5. The impact angle of the bullet relative to the blade was set by the angle-adjusting fixture. The deviation of the incidence angle was within 1 degree. The accuracy of the impact point was ensured by the sighting instrument of the ballistic gun. The muzzle is 1.5 m away from the impacted point of the blade. The speed of the bullet is controlled to be 650 +/− 20 m/s.

## 3. Numerical Simulation of Composite Blade under Ballistic Impact

### 3.1. Finite Element Model and Boundary Conditions

**Figure 6:**
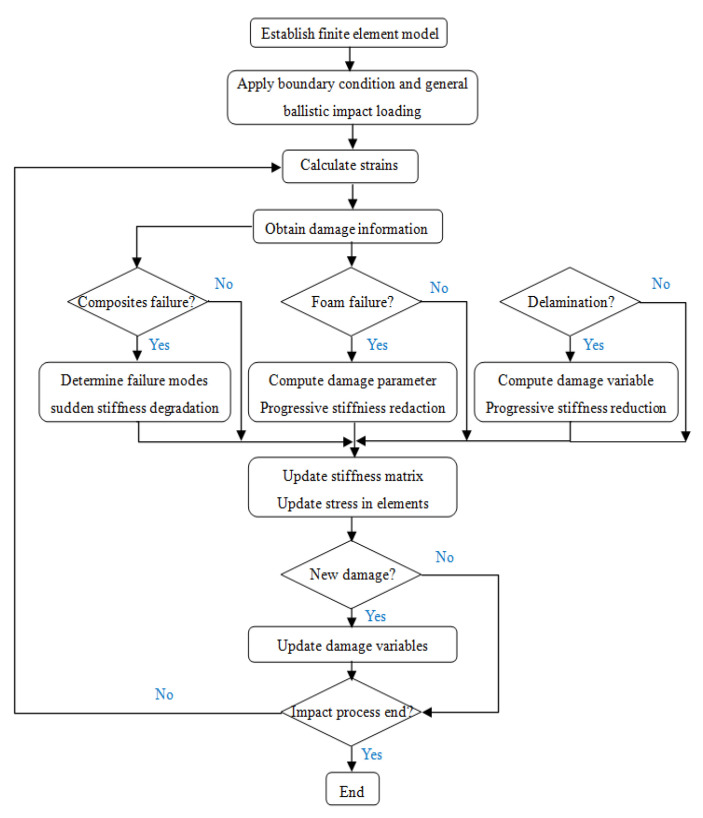
Analysis and calculation flow.

**Figure 7:**
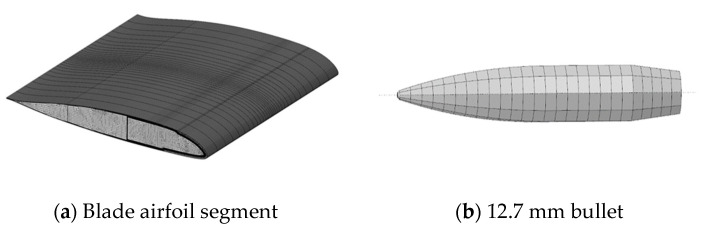
Schematic of the finite element.

In order to study the failure mechanism of the composite blade under ballistic impact, a numerical simulation model including skin, spar, foam filling, and reinforcing ribs was established using Abaqus software (Version 6.14); the analysis process is shown in Figure 6. The finite element model of the bullet is established according to the actual size of the bullet and is regarded as a rigid body (Figure 7). The 8-node hexahedral linear reduced integration element C3D8R was selected to analyze the damage characteristics of the composite blade to save computational time. The bonding units COH3D8 were introduced between the skin and foam interface and each layer of skin, considering the simulation of the delamination of composite blades under high-speed impact. The mechanical properties of bonding units are provided in Table 2. PMI adopts the crushable foam model, and the material properties are shown in Table 3.

**Figure 8:**
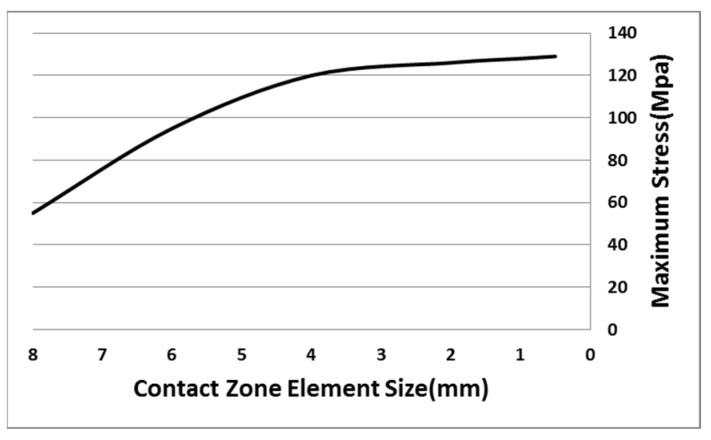
Element mesh size dependency analysis.

During the process of high-speed impact by the bullet, the deformation of the blade mainly occurs at the contact zone between the bullet and the blade. The grid density decreases from the impact zone to the edge of the blade. This kind of grid not only ensures the accuracy of the numerical results but also improves the computational efficiency of the model. The element mesh size dependency analysis was conducted on the impact contact area as shown in Figure 8, and based on the analysis results, a element size of 1mm was selected in the impact contact area.

**Figure 9:**
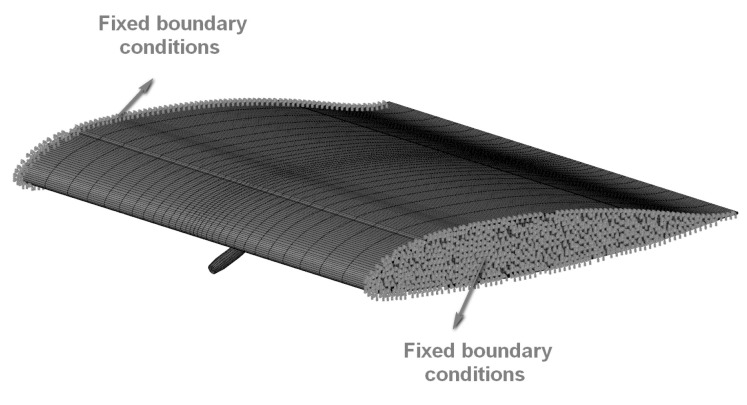
The fixed boundary conditions.

The fixed boundary conditions (shown in Figure 9) are applied to the two ends of the composite blade, and all the degrees of freedom are constrained to simulate the clamping fixation of the impact experiment. The initial velocity of the bullet is set to 650 m/s, which is the same as the actual experiment condition.

The contact response between the bullet and the blade is simulated by surface contact. The contact force is calculated by a finite sliding penalty contact algorithm.

### 3.2. Material Damage Model

Capturing the damage modes seen in the experiment is a key requirement of the model. Selecting proper failure criteria is essential to accurately detecting the onset of material damage.

#### 3.2.1. Damage Model of Composite Materials

The failure strength of composites is mainly related to the properties of materials, stress state, strain state, and strain energy under loading. The failure modes of composite materials are mainly as follows: fiber tensile failure, fiber compression failure, matrix tensile failure, and matrix compression failure. The Hashin failure criterion is one of the classical failure criteria for composite materials. It is based on the stress failure criterion. According to the different failure mechanisms produced by tensile and compressive failure, the failure of the fiber and matrix of composite materials is classified into tensile and compressive failure [24,25].

There are two kinds of composite materials used in the blade design of this work. One is the warp-knitting fabric used for the spar, which belongs to the unidirectional fiber composite material. The other is the composite plain fabric used for skin, which belongs to a bidirectional fiber-reinforced composite material. The failure criteria used for the two composites are different.

The failure criteria used for spar are as follows:

Fiber tensile failure ($\sigma_{1}\geq 0)$

(1) $$({\frac{\sigma_{1}}{X_{T}})}^{2}+({\frac{\sigma_{12}}{S_{12}})}^{2}\geq 1$$

Fiber compression failure $\left(\sigma_{1}<0\right)$

(2) $$({\frac{\sigma_{1}}{X_{C}})}^{2}\geq 1$$

Matrix tensile failure $\left(\sigma_{2}+\sigma_{3}\geq 0\right)$

(3) $$({\frac{\sigma_{2}+\sigma_{3}}{Y_{T}})}^{2}+({\frac{\sigma_{12}}{S_{12}})}^{2}+({\frac{\sigma_{13}}{S_{13}})}^{2}\geq 1$$

Matrix compression failure $\left(\sigma_{2}+\sigma_{3}\geq 0\right)$

(4) $${\left(\frac{\sigma_{2}+\sigma_{3}}{Y_{C}}\right)}^{2}\geq 1$$

The failure criteria used for skin are as follows: 

Warp tensile failure ($\sigma_{1}\geq 0$)

(5) $$({\frac{\sigma_{1}}{X_{T}})}^{2}+\alpha ({\frac{\sigma_{12}}{S_{12}})}^{2}+\alpha ({\frac{\tau_{13}}{S_{13}})}^{2}\geq 1$$

Warp compression failure ($\sigma_{1}<0$)

(6) $$({\frac{\sigma_{1}}{X_{C}})}^{2}\geq 1$$

Weft tensile failure ($\sigma_{2}\geq 0$)

(7) $$({\frac{\sigma_{2}}{Y_{T}})}^{2}+\alpha ({\frac{\sigma_{12}}{S_{12}})}^{2}+\alpha ({\frac{\tau_{23}}{S_{23}})}^{2}\geq 1$$

Weft compression failure ($\sigma_{2}<0$)

(8) $$({\frac{\sigma_{2}}{Y_{C}})}^{2}\geq 1$$

Matrix tensile failure ($\sigma_{3}\geq 0$)

(9) $$({\frac{\sigma_{3}}{Z_{T}})}^{2}+({\frac{\sigma_{13}}{S_{13}})}^{2}+({\frac{\sigma_{23}}{S_{23}})}^{2}\geq 1$$

Matrix compression failure ($\sigma_{3}\geq 0$)

(10) $$({\frac{\sigma_{3}}{Z_{C}})}^{2}\geq 1$$

where $X_{T}$, $X_{C}$, $Y_{T}$, and $Y_{C}$ represent the longitudinal tensile strength, the transverse tensile strength, and the compressive strength of the fiber on the surface of the composite material, respectively. $Z_{T}$ and $Z_{C}$ are the tensile strength and the compressive strength of the matrix. $S_{12}$, $S_{13}$, and $S_{23}$ are in-plane shear strength and inter-laminar shear strength, respectively, and α is the shear correction factor.

The failure of composite material occurs through the whole high-speed penetration process. When the composite material is subjected to a high-speed dynamic impact load, the bearing capacity of the damage direction will be reduced. But not all the failures directly. Therefore, a reasonable stiffness reduction scheme should be adopted to properly reduce the stiffness. When both the composite fiber and the matrix lose their load-bearing capacity, the element will be deleted from the finite element model. The stiffness reduction scheme [26] of this paper is shown in Table 4.

#### 3.2.2. Damage Model of a Cohesive Unit

Delamination failure is also an important failure mode for composites under high-speed impact loads. Since cohesive elements are widely used to model adhesives between two components, interfacial debonding, gaskets, and small adhesive patches. In this paper, the cohesive elements are inserted into the interfaces between the adjacent layers inside the composite skin and between the skin and the filled foam, which aims at effectively simulating the delamination mode under ballistic impact. 

**Figure 10:**
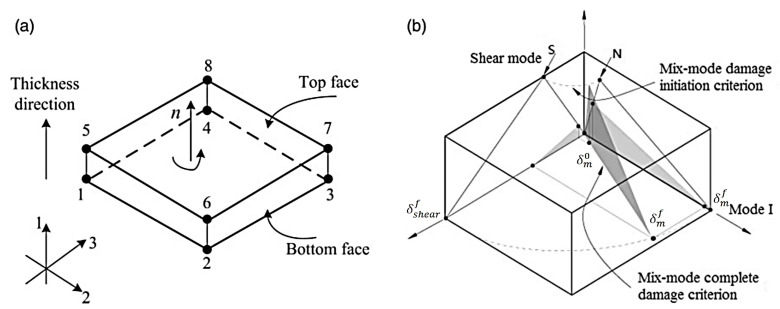
Cohesive zone model of interface: (**a**) coordinate system of cohesive element and (**b**) mix-mode bilinear constitutive model of cohesive element.

The coordinate system of cohesive elements is displayed in Figure 10a. It is assumed that only a normal stress $t_{1}$ and two shear stresses $t_{2}$,$t_{3}$ exist, since the cohesive interface is extremely thin. The interface behavior is governed by a bilinear traction-separation law, which adopts an initially linear elastic response followed by the linear evolution of damage (Figure 10b).

The initial elastic response of the cohesive elements can be expressed as

(11) $$\left\{\begin{array}{c} t_{1}\\ t_{2}\\ t_{3} \end{array}\right\}=\left[\begin{array}{ccc} k_{1} & 0 & 0\\ 0 & k_{2} & 0\\ 0 & 0 & k_{3} \end{array}\right]\left\{\begin{array}{c} \delta_{1}\\ \delta_{2}\\ \delta_{3} \end{array}\right\}$$

where $k_{i}$ is the initial stiffness and $\delta_{i}$ is the separation displacement.

The quadratic nominal stress criterion is used as the damage initiation of the cohesive elements [26]:

(12) $$({\frac{\langle t_{1}\rangle }{N})}^{2}+({\frac{t_{2}}{S})}^{2}+({\frac{t_{3}}{T})}^{2}=1$$

where N, S, and T are normal and two tangential interface strengths, respectively.

Normal compressive stress does not contribute to the damage initiation of cohesive elements. Hence, the Macaulay operator in Equation (12) is defined by

(13) $$\langle a\rangle =\left\{\begin{array}{cc} a & a>0\\ 0 & a\leq 0 \end{array}\right.$$

The material stiffness needs to be degraded once the initiation criterion is satisfied. After damage initiation, a scalar damage variable d is used to represent the linear damage evolution of the interface:

(14) $$d=\frac{\delta_{m}^{f}(\delta_{m}-\delta_{m}^{0})}{\delta_{m}(\delta_{m}^{f}-\delta_{m}^{0})}\;\left(d\in \left[0,1\right]\right)$$

Here, $\delta_{m}$ is the equivalent displacement determined by Equation (15), including normal and tangential displacement. $\delta_{m}^{0}$ represents the equivalent displacement at the beginning of interface damage. $\delta_{m}^{f}$ is the equivalent displacement at the complete failure of the interface.

(15) $$\delta_{m}=\sqrt{{\langle \delta_{1}\rangle }^{2}+{\delta_{2}}^{2}+{\delta_{3}}^{2}}$$

The damage variable d is introduced into the constitutive relationship of the cohesive unit:

(16) $$\left\{\begin{array}{c} \hat{t_{1}}\\ \hat{t_{2}}\\ \hat{t_{3}} \end{array}\right\}=\left[\begin{array}{ccc} 1-d\frac{\langle t_{1}\rangle }{t_{1}} & 0 & 0\\ 0 & 1-d & 0\\ 0 & 0 & 1-d \end{array}\right]\left[\begin{array}{ccc} k_{1} & 0 & 0\\ 0 & k_{2} & 0\\ 0 & 0 & k_{3} \end{array}\right]\left\{\begin{array}{c} \delta_{1}\\ \delta_{2}\\ \delta_{3} \end{array}\right\}$$

Here, the symbol (^) denotes actual stress.

#### 3.2.3. Foam Damage Model

**Figure 11:**
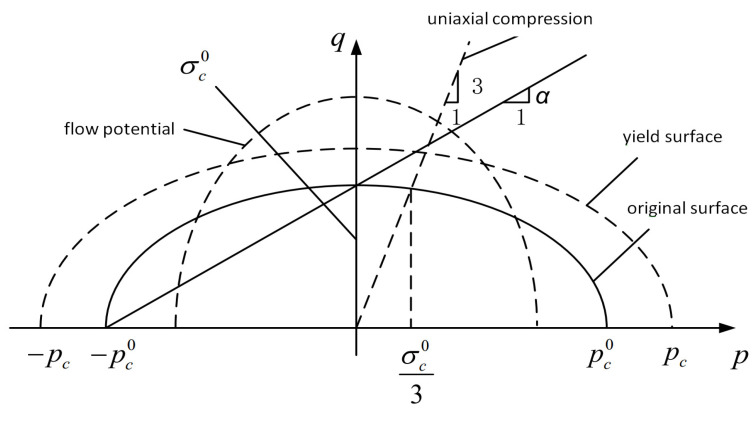
The compression process of the model foam of each isotropic.

The crushable foam model [27] is used to solve the problems of material strength and damage by making the filled foam equivalent to macroscopic homogeneous continuous material. In this paper, as shown in Figure 11, an isotropic hardening compressible foam model is used to describe the mechanical behavior of the filled foam under a high-speed impact load. The yield stress expression is as follows: it is an ellipse centered at the stress plane p–q origin and evolves in a self-similar manner.

(17) $$F=\sqrt{q^{2}+\beta^{2}{\left(p-p_{0}\right)}^{2}}-B=0$$

Here, p is hydrostatic pressure; q is Mises stress; β is the ellipse shape factor; B is ellipse q axis length; and $p_{0}$ is an ellipse major axis center.

Shear plugging is the main damage mode of foam under high-speed impact load [28], so the shear damage criterion is adopted as the damage criterion. This criterion is a high strain rate deformation design, using equivalent plastic strain as the failure measure, and can be used in conjunction with Mises or Johnson–Cook plastic models. The shear stress ratio in this guideline is defined as follows:

(18) $$\theta_{S}=\frac{q+k_{s}p}{\tau_{max}}$$

Here, q is Mises equivalent stress; $k_{s}$ is a material parameter; the value of closed-cell foam is 0 [27]; p is hydrostatic pressure; and $\tau_{max}$ is the maximum shear stress.

## 4. Results and Discussion

### 4.1. Experiment Result Analysis

**Figure 12:**
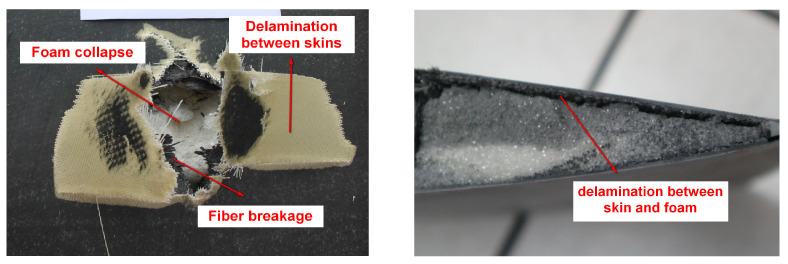
Ballistic failure model of the composite blade.

As can be seen from the ballistic results of Figure 12, the bullet passes through the blade airfoil section quickly, leaving holes in the upper and lower airfoil surfaces. The failure of composite material blades is mainly divided into three parts. Part one is that the composite skin is torn apart, and the main failure mode is fiber breakage. Part two is the delamination caused by bonding failure, including the delamination between skins and between skins and foams. Part three is the foam collapse, which is in the shape of powders and loses its bearing capacity.

### 4.2. Model Verification

#### 4.2.1. Comparison of Damage Morphology

**Figure 13:**
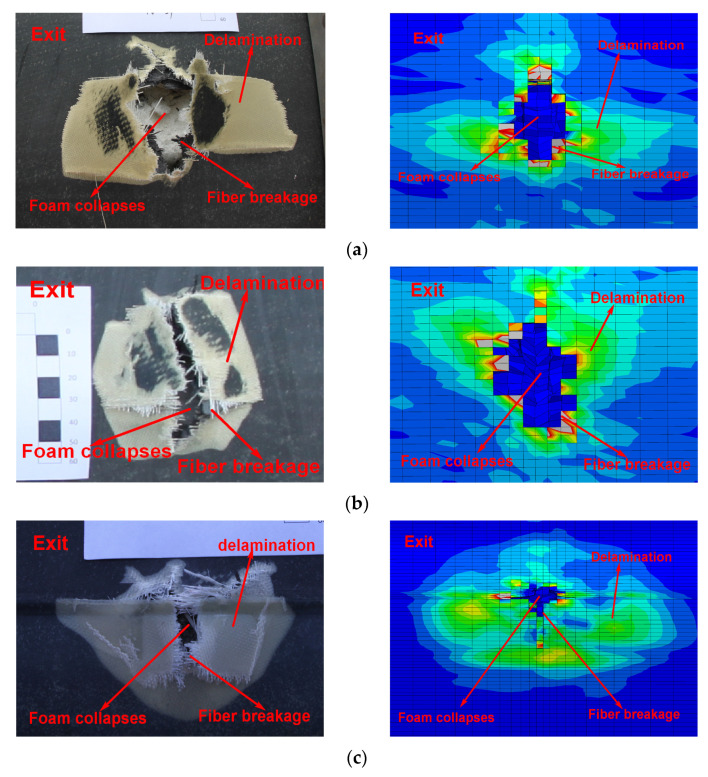
Comparison of damage morphology: (**a**) case 1, (**b**) case 2, and (**c**) case 3.

**Figure 14:**
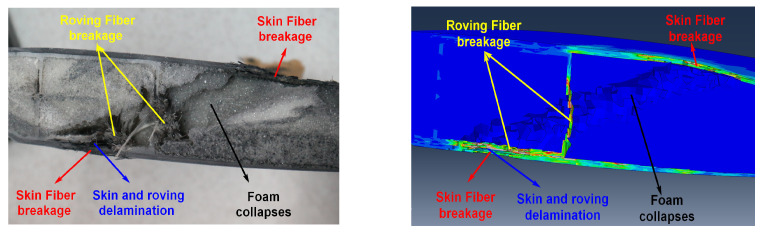
Cross-sectional damage comparison.

Figure 13 and Figure 14 show the comparison between the damage experimental results and numerical results at the exit of the blade after being impacted by a bullet under three experimental conditions. The simulation results can predict the damage mode of the blade. According to the experimental results, the damage to the blade is mainly in the form of skin delamination, fiber breakage, and foam collapse. The damage characteristics at the exit of the skin obtained by simulation are in good agreement with the results obtained by the ballistic experiment.

#### 4.2.2. Comparative Analysis of the Damage Range

**Figure 15:**
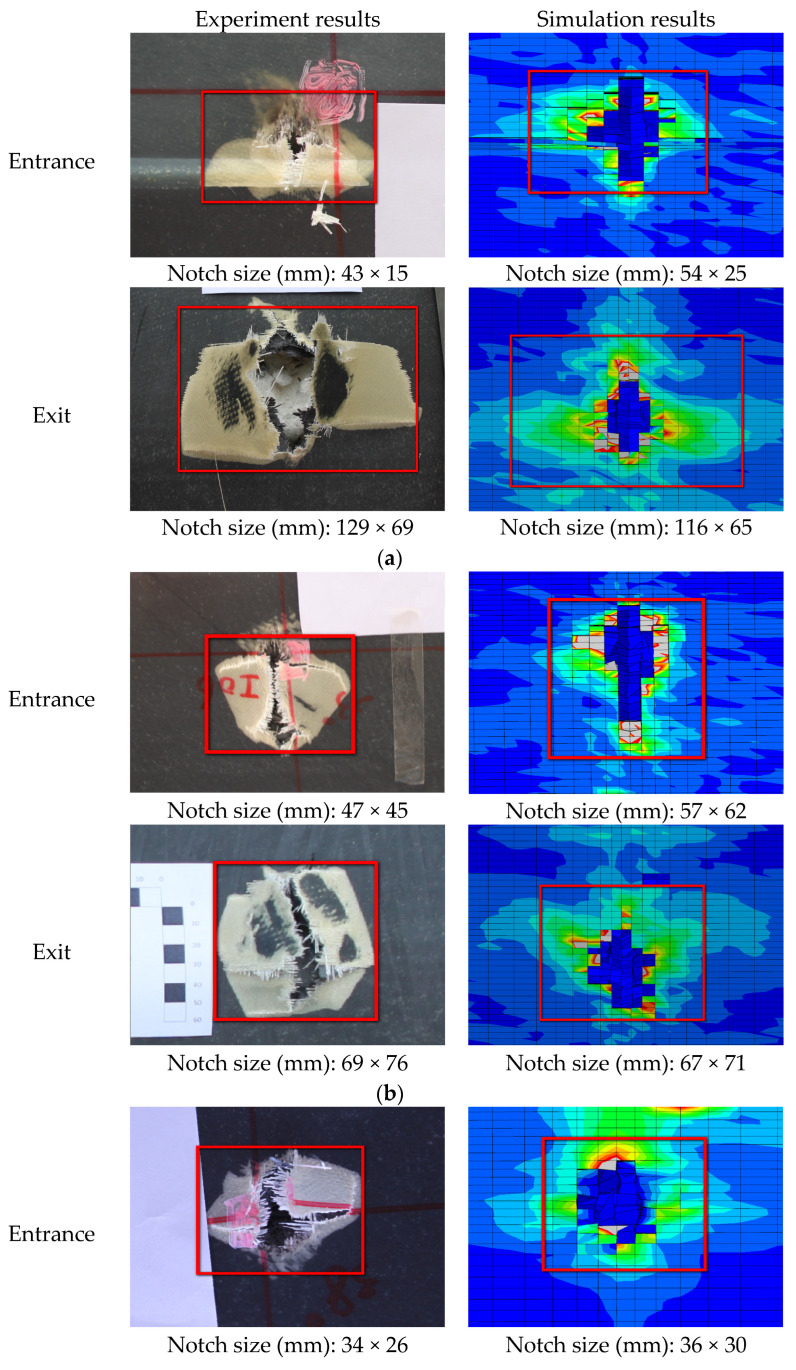

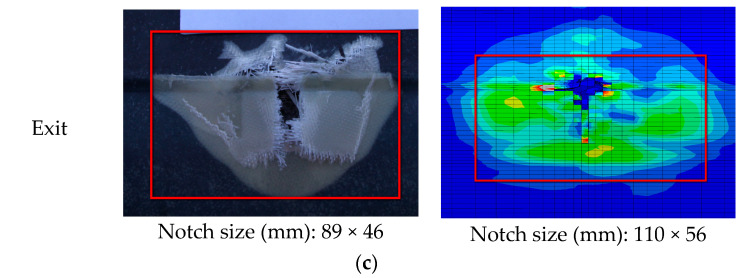
Comparison of damage range between experiment and simulation results: (**a**) case 1, (**b**) case 2, and (**c**) case 3.

As shown in Figure 15, the size of the damage notch in the simulation analysis and the ballistic experiment are compared. The damage notches on the surface of the skin show that the simulation results are in good agreement with the experiment results. The numerical simulation model can accurately predict the damage range of composite blades subjected to ballistic impact.

## 5. Analysis of the Bullet Penetration Process

**Figure 16:**
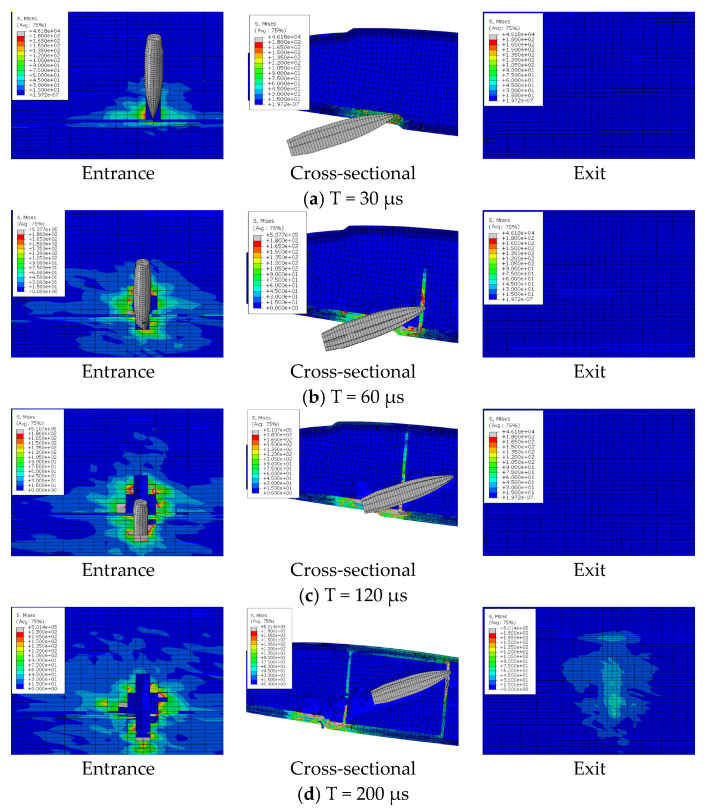

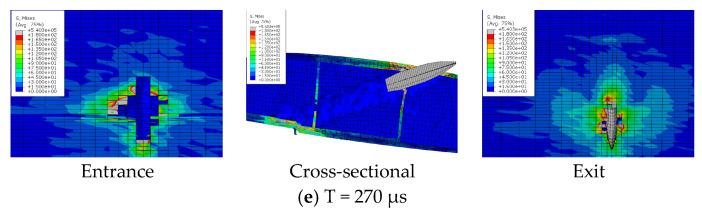
Ballistic penetration process of case 1.

**Figure 17:**
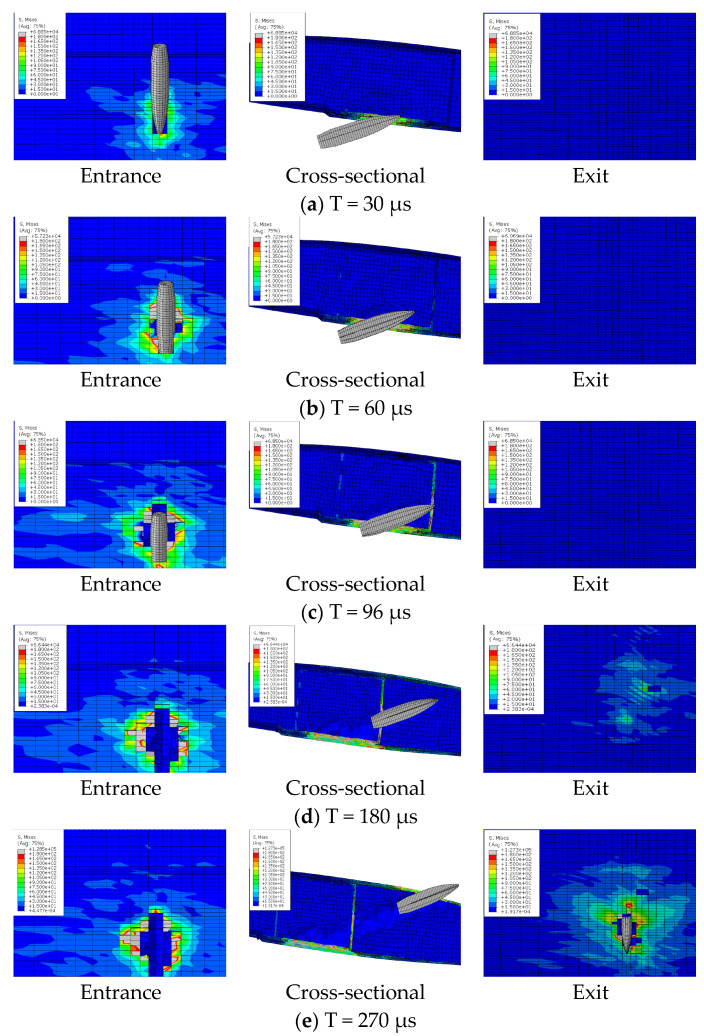
Ballistic penetration process of case 2.

**Figure 18:**
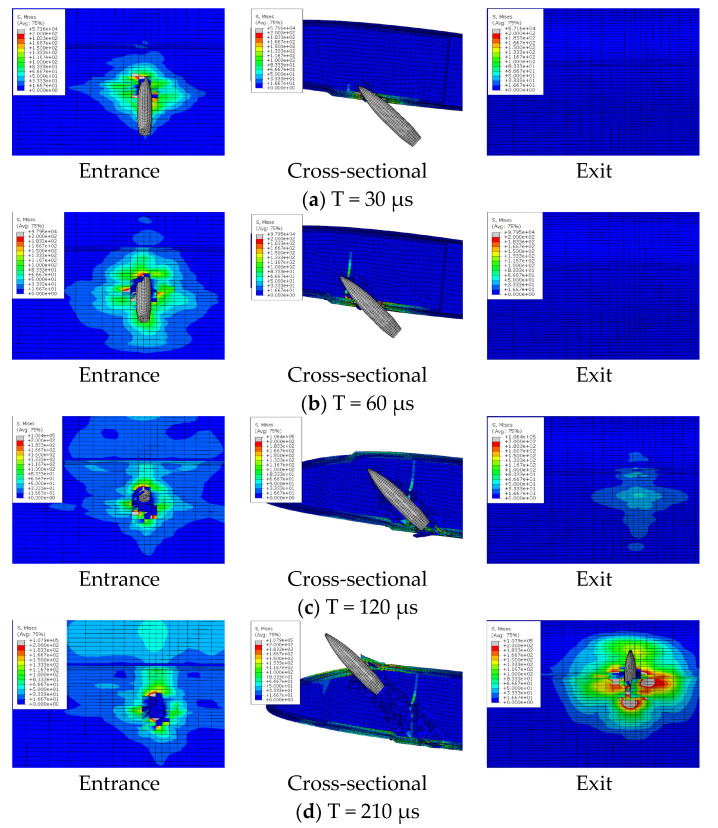
Ballistic penetration process of case 3.

The ballistic process of composite blades is a transient dynamic process. The damage to the blade is determined by the local properties of the structure near the impact zone. There are different failure modes of the composite blade during ballistic penetration, including fiber tensile failure, delamination failure, and foam collapse. Through the finite element model, the deformation, damage expansion, and delamination of the blade under the bullet penetration process can be analyzed in detail. Figure 16, Figure 17 and Figure 18 show the penetration process of bullets under three experimental conditions. With a speed of 650 m/s, the duration of the bullet penetration process is less than 400 μs. During the projectiles, different damage modes occur, promote, and couple in succession.

### 5.1. Experiment Result Analysis

The bullet penetration process under three conditions can be divided into four stages: incident skin failure stage, foam failure stage, reinforced rib failure stage, and exit skin failure stage.

(1) The incident skin failure stage is mainly the failure damage and damage expansion of the lower wing surface skin. The bullet with high kinetic energy exerts strong pressure on the skin, but due to the dispersion of the load by the foam core, the skin at the entrance experiences local shearing-type failure only in the contact area. Meanwhile, the foam can be observed to produce a small range of collapse, which is due to the fiber block of the fractured skin at the entrance due to the extrusion under the push of the projectile.

(2) The foam sandwich core failure stage is mainly caused by foam press damage. The material stress in a very small strain range increases rapidly. The foam generates collapse and fails to damage. The ballistic hole of the foam core part is almost as wide as the bullet body at this time. It indicates that the failure mode is mainly local shear fracture.

(3) The failure stage of the reinforcing rib is mainly the destruction and damage expansion of the reinforcing rib and foam. The bullet passes through the foam to impact the reinforcing rib, and the reinforcing rib bears strong tensile stress. The failure mode is fiber breakage.

(4) The failure stage of the skin at exit is mainly the failure and damage expansion of the skin. The skin at the exit deformed greatly in the process of destruction before the bullet contacted the skin. Due to the accumulation of foam on the front side of the ballistic and extruding the outer skin and the transmission of impact stress waves, the skin at exit already has protrusion deformation, which leads the back fiber to bear strong compressive stress. The stress state leads to the origin of the outer skin damage, and the failure mode is different from the incident skin. The maximum stress appears on the outer surface. The skin begins to break from the outermost side, and the fiber tensile fracture is the main damage.

Because the skin at the exit has certain stress and deformation before the bullet arrives, and stress is further increased and diffused when the bullet penetrates the skin, the damage size of the skin at the exit is much larger than that of the skin at the entrance. As can be seen from Figure 13, in the three projectile conditions, the ballistic damage size of the exit port can reach six times the ballistic damage size of the entrance port.

As can be seen from the impact penetration paths of the three experimental conditions, the bullet always passes obliquely through the leading and trailing edges of the blade profile. Due to the large bullet size (diameter 12.7 mm, length 65 mm) and blade size (chord length 500 mm, airfoil thickness 60 mm), one impact will only cause local damage to the upper and lower airfoil surfaces. The distribution design of the upper and lower airfoil surfaces should be considered in the design of the main bearing structure. The spar and the skin-reinforcing cloth should not be concentrated on the front edge of the paddle. To avoid large damage to the bearing structure caused by one bullet, the spar and the skin reinforcing cloth should be distributed to the upper wing surface and the lower wing surface as much as possible. More reinforcing ribs bearing shear load should be designed in the section to form multiple closed cavities to ensure that all shear load-bearing structures will not be damaged by one shot.

### 5.2. The Transmission of Stress Waves during Bullet Penetration

**Figure 19:**
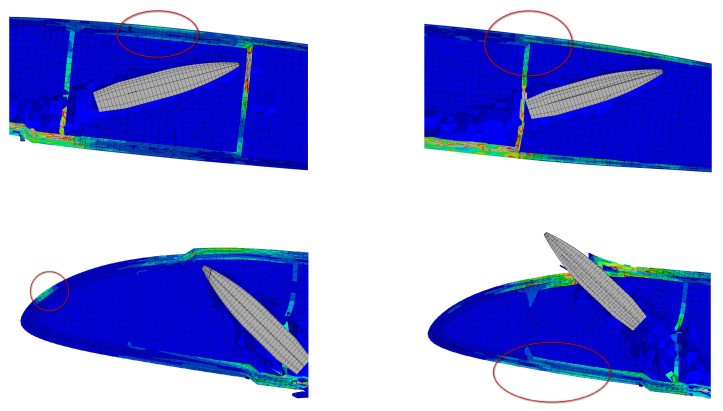
Stress on the non-impact path.

During the bullet penetration process, the blade structure experiences both the direct impact effect on the bullet penetrating path and the transmission of stress waves caused by the high-speed impact response within the blade. As shown in Figure 19, these stress waves propagate through spars, reinforcing ribs, and other components, resulting in significant stress within the non-shock penetration region. While the stress values may not immediately cause structural damage or invalidation, it is important to consider that the blade is in a non-loaded state during simulation analysis. Therefore, under actual operational conditions, the loads may cause damage outside the trajectory.

### 5.3. Analysis of Factors Influencing the Range of Ballistic Damage

#### 5.3.1. Analysis of Incident Angles

**Figure 20:**
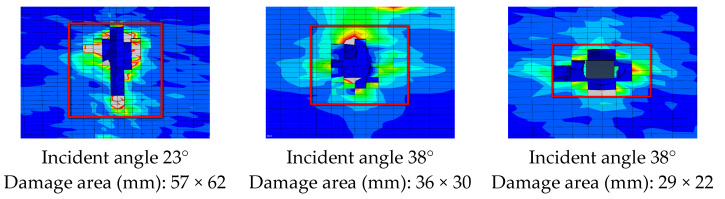
Comparison of the damage at the exit of different incident angles.

Cases 2 and 3 are in the same incident position but have different incident angles. Due to the different impact angles, the damage to the incident port is different (shown in Figure 20). From the angle of the incident port damage size, the larger the incident angle formed with the skin plane, the greater the damage caused. Relatively speaking, the vertical entry results in minimal damage.

**Figure 21:**
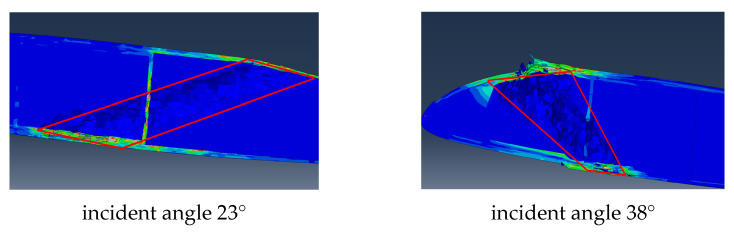
Comparison of trajectory damage at different impact angles.

Due to the curved surface shape and complex internal structure of the blade, it is not similar to a foam sandwich board. The interior is filled with foam but features structures such as spars and reinforcing ribs. Therefore, different incident angles directly lead to different ballistic paths. As a result, the damage caused by different load-bearing structures along the path varies (shown in Figure 21). In case 2, the incident angle is obliquely pointed towards the rear edge, so the ballistic path of the bullet is only the reinforcing rib in the middle of the blade section. This results in a small damage range during the bullet impact process. On the other hand, in case 3, the incident angle points to the position of the front edge spar, which is the main load-bearing structure, causing more damage and a wider trajectory.

#### 5.3.2. Analysis of Structural Characteristics

**Figure 22:**
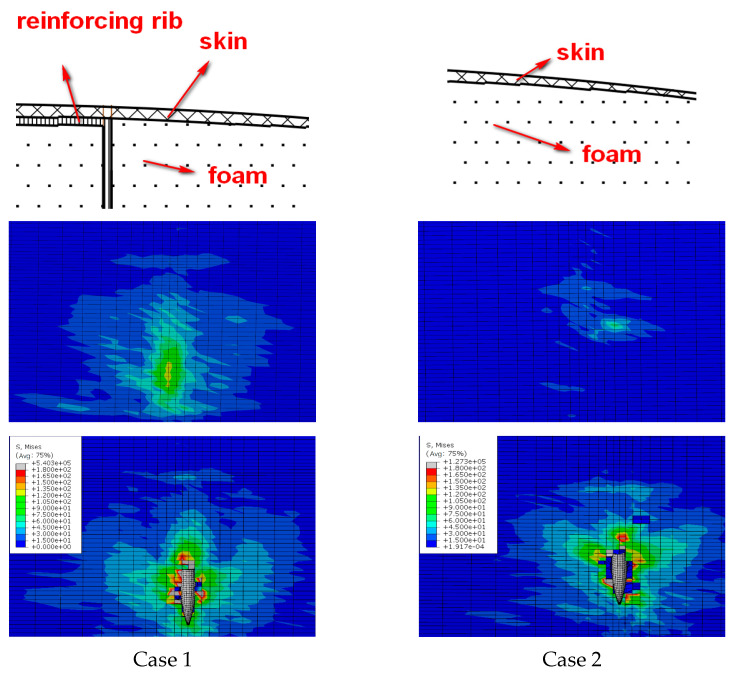
Comparison of ballistic damage at different structural characteristics.

As can be seen from Figure 22, the incident angle in case 1 and case 2 is the same, but the skin damage range of the exit opening is quite different. The damage size of the exit opening in case 1 is 116 mm × 65 mm, while it is 67 mm × 71 mm in case 2.

The comparative analysis of the structure at the exit in case 1 and case 2 shows that the structure at the exit of case 1 consists of skin and spar. The structure at the exit of case 2 consists of only skin. The structure at the exit of case 1 has greater stiffness than that of case 2. Therefore, in the structure at the exit of case 1, before touching the bullet, the stress caused by foam extrusion and stress wave transmission is greater, the range is wider, and the bullet can absorb more energy during bullet penetration, so the bullet damage is greater. That is to say, the more bearing structure and stiffness near the exit, the larger the range of ballistic damage.

## 6. Conclusions

In this paper, the damage characteristics of the composite rotor blade under ballistic impact are studied by experiments. A numerical simulation model considering a three-dimensional progressive damage failure model that can simulate and analyze the impacted blade ballistic damage is established. The simulation results are compared with the experimental results to verify the accuracy of the presented numerical simulation model. Furthermore, the ballistic performance of composite blades impacted by different incident angles and different locations is systematically studied. The conclusions are as follows:

(1) The main damage forms of the impacted composite blade by the bullet are fiber breakage, delamination, and foam collapse. The damage size and characteristics of the numerical results, which are calculated by the established numerical simulation model, are consistent with the experimental results under different ballistic conditions.

(2) Through the bullet penetration process, the form and mechanism of blade damage vary. The damage of skin at the entrance is mainly local shear failure caused by short-term strong pressure. Foam failure is mainly caused by the rapid increase of stress in a very small strain range. The damage of the reinforced rib and the skin at the exit is mainly due to the tensile fracture of the back fiber caused by strong compressive stress. This leads to the damage size of the exit being generally larger than that of the entrance.

(3) The damage size of the impacted blade at the entrance is mainly related to the incident angle of the bullet. The larger the incident angle, the smaller the impact damage size at the entrance. The impact damage size of the exit is mainly related to the structural characteristics of the structure. The more load-bearing structures and the greater the stiffness at the exit, the larger the impact damage size at the exit.

(4) Impact by one bullet will only cause damage to the local positions of the upper and lower airfoil surfaces. Therefore, in the design of the blade structure, the distribution design of the main load-bearing structures along the airfoil surfaces should be considered, and more reinforcing ribs for bearing shear load should be designed to form a plurality of closed cavities to reduce the damage to the main bearing structure of the blade impacted by one bullet.

(5) Due to the extrusion and the transmission of the stress wave of the foam, the skin at the exit has great stress and deformation, resulting in greater damage at the exit. The material with lower consistency, such as honeycomb material, should be selected as far as possible as the filling material of the rotor blade. This makes the bullet pass through the material quickly without causing material accumulation and extrusion, thereby reducing the damage to the skin at the exit. 

We have shown that the proposed numerical model is effective in predicting the ballistic performance and analyzing the failure mechanism of the composite blade under various ballistic impact conditions. It provides a theoretical basis and reference for the anti-ballistic design and ballistic performance analysis of composite blades.

## Figures and Tables

**Table 1:** Material properties of composite materials.

Property	EW250F/Epoxy	CF3052/Epoxy	S4C10-800/Epoxy	Property	EW250F/Epoxy	CF3052/Epoxy	S4C10-800/Epoxy
$E_{1}\;(GPa)$	25	50.4	57	$X_{T}\;(Mpa)$	380	520	1350
$E_{2}\;(GPa)$	25	50.4	13	$X_{C}\;(Mpa)$	380	380	700
$E_{3}\;(GPa)$	3	6.8	13	$Y_{T}\;(Mpa)$	380	520	50
$\nu_{12}$	0.14	0.08	0.3	$Y_{C}\;(Mpa)$	380	380	200
$\nu_{13}$	0.2	0.35	0.3	$Z_{T}\;(Mpa)$	33.6	33.6	50
$\nu_{23}$	0.2	0.35	0.33	$Z_{C}\;(Mpa)$	93.6	93.6	200
$G_{12}\;(GPa)$	4.5	3.13	5.5	$S_{12}\;(Mpa)$	80	80	52
$G_{13}\;(GPa)$	2.4	2.78	5.5	$S_{13}\;(Mpa)$	49	62	52
$G_{23}\;(GPa)$	2.4	2.78	5.5	$S_{23}\;(Mpa)$	49	62	50

**Table 2:** Mechanical properties of bonding units.

Property	Value	Property	Value
E (GPa)	5	$t_{s}^{0}\;(MPa)$	30
G (GPa)	5	$G_{n}^{c}\;(N/mm)$	0.6
$t_{n}^{0}\;(MPa)$	30	$G_{s}^{c}\;(N/mm)$	2.1

**Table 3:** Material properties of PMI.

Property	Value	Property	Value
E (GPa)	92	δ(%)	30
G (GPa)	29	$\rho \;(kg/m^{3})$	75
$\sigma_{t}\;(Mpa)$	2.8	ν	0.3
$\sigma_{c}\;(Mpa)$	1.5	$k_{s}$	0
S (MPa)	1.3	$\theta_{s}$	1.5

**Table 4:** Composite stiffness reduction scheme.

Material	Damage Model	Stiffness Reduction Scheme
Spar	Fiber failure	E_1_ = 0.1E_1_
	Matrix failure	E_2_ = 0.2E_2_, E_3_ = 0.2E_3_, G_12_ = 0.2G_12_, G_23_ = 0.2G_23_
Skin	Warp failure	E_1_ = 0.1E_1_, G_12_ = 0.1G_12_, G_13_ = 0.1G_13_, υ_12_ = 0.1υ_12_, υ_13_ = 0.1υ_13_
	Weft failure	E_2_ = 0.1E_2_, G_12_ = 0.1G_12_, G_23_ = 0.1G_23_, υ_12_ = 0.1υ_12_,υ_23_ = 0.1υ_23_
	Matrix failure	E_3_ = 0.2E_3_, G_12_ = 0.2G_12_, G_23_ = 0.2G_23_

## Data Availability

Data are contained within the article.

## Dual-use Research Statement

We have made the following statements to our paper titled “Performance and Damage Study of Composite Rotor Blades under Impact”.

Ø Explanation of Potential Risks: Our paper examines the performance of composite rotor blades under impact. The research is limited to providing some theoretical and experimental support for the composite rotor blades under impact only and does not pose a threat to public health or national security.
Ø Evaluation of Benefits to the General Public: Our research is limited to the academic field, which is beneficial to the development of composite rotor blades under impact. There is no risk to the general public.
Ø Compliance with Laws: As an ethical responsibility, we strictly adhere to relevant national and international laws about dual-use research. And we have considered and adhered to these regulations in our paper.
